# Distribution of Scholarships

**Published:** 1884-03

**Authors:** 


### DISTRIBUTION OF SCHOLARSHIPS.

We are in receipt of "A Reply" to Louisville Medical College, by Dr. D. W. Yandell (reprint from the *American Practitioner*), which arraigns the Louisville Medical College for sending "scholarships" by its faculty and attaches to students, promiscuously, both North and South.

Several years ago, the same school indulged in these unworthy methods, to the extent that the profession was shocked by its enormity, and demanded a halt. The school then promised to cease its evil doings and to be governed by the laws of the Association of Medical Colleges, but it would appear that she has again fallen into her former evil ways. That the "scholarships," as they are denominated by those sending them out, are sent by members of the faculty, there can be no doubt, as we have seen more than one, and have heard of many others received by medical students in Georgia and Alabama. This practice of medical colleges, which we hope and believe is at present confined to very narrow limits, of attempting to swell the number of their classes by such unprofessional methods should cease entirely. It may for a few years swell the number on their benches, but at last will result in the downfall of their institution, as in the Louisville Medical College a few years ago, from which descent it is slow to rise, as the present faculty has long since found out. This of itself, it would appear, should deter them from such evil practices, if they looked alone to the future success of the institution, without taking into consideration the moral aspect of the subject, or the law of medical colleges which they propose to be governed by.

The best policy for every medical school is to stand squarely on its own merits, and never descend to illegitimate methods to swell the number of students in attendance. Fair dealing, after all, is the best policy in medical teaching, as well as in every other legitimate business.

